# Aqua­tris[6-(3,5-dimethyl-1*H*-pyrazol-1-yl)picolinato]-κ^6^ *N*,*N*′,*O*;κ*O*-dysprosium(III) trihydrate

**DOI:** 10.1107/S1600536808001943

**Published:** 2008-01-25

**Authors:** Zhao Kai, Feng Yu, Xian-Hong Yin, Zhu Jie, Cui-Wu Lin

**Affiliations:** a College of Chemistry and Ecological Engineering, Guangxi University for Nationalities, Nanning 530006, People’s Republic of China; b College of Chemistry and Chemical Engineering, Guangxi University, Nanning 530004, People’s Republic of China

## Abstract

In the title complex, [Dy(C_11_H_10_N_3_O_2_)_3_(H_2_O)]·3H_2_O, the Dy^III^ atom is coordinated by four N atoms and four O atoms derived from three tridentate deprotonated 6-(3,5-dimethyl-1*H*-pyrazol-1-yl)picolinate ligands and one water mol­ecule. The complex and solvent water mol­ecules are linked together *via* O—H⋯O, O—H⋯N, C—H⋯O and C—H⋯π hydrogen-bonding inter­actions, forming a three-dimensional network structure.

## Related literature

For related literature, see: Zhao *et al.* (2008 ▶); Yin *et al.* (2007 ▶); Baggio *et al.* (2003 ▶).
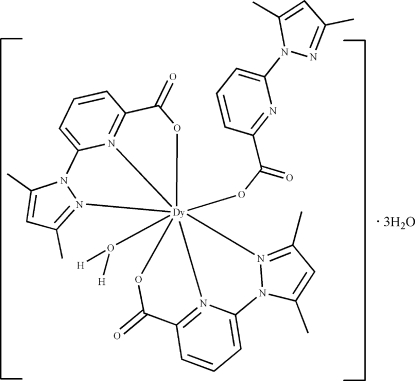


## Experimental

### 

#### Crystal data

[Dy(C_11_H_10_N_3_O_2_)_3_(H_2_O)]·3H_2_O
*M* *_r_* = 883.22
Monoclinic, 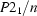

*a* = 15.4709 (18) Å
*b* = 12.8466 (12) Å
*c* = 18.543 (2) Å
β = 99.741 (2)°
*V* = 3632.3 (7) Å^3^
*Z* = 4
Mo *K*α radiation
μ = 2.13 mm^−1^
*T* = 298 (2) K
0.30 × 0.24 × 0.17 mm

#### Data collection

Siemens SMART CCD area-detector diffractometer
Absorption correction: multi-scan (*SADABS*; Sheldrick, 1996 ▶) *T*_min_ = 0.568, *T*_max_ = 0.714
17074 measured reflections
6277 independent reflections
4009 reflections with *I* > 2σ(*I*)
*R*_int_ = 0.063

#### Refinement

*R*[*F* ^2^ > 2σ(*F* ^2^)] = 0.050
*wR*(*F* ^2^) = 0.118
*S* = 1.04
6277 reflections
478 parameters
H-atom parameters constrained
Δρ_max_ = 1.45 e Å^−3^
Δρ_min_ = −0.93 e Å^−3^

### 

Data collection: *SMART* (Siemens, 1996 ▶); cell refinement: *SAINT* (Siemens, 1996 ▶); data reduction: *SAINT*; program(s) used to solve structure: *SHELXS97* (Sheldrick, 2008 ▶); program(s) used to refine structure: *SHELXL97* (Sheldrick, 2008 ▶); molecular graphics: *SHELXTL* (Sheldrick, 2008 ▶); software used to prepare material for publication: *SHELXTL* and *PLATON* (Spek, 2003 ▶).

## Supplementary Material

Crystal structure: contains datablocks I, global. DOI: 10.1107/S1600536808001943/si2070sup1.cif

Structure factors: contains datablocks I. DOI: 10.1107/S1600536808001943/si2070Isup2.hkl

Additional supplementary materials:  crystallographic information; 3D view; checkCIF report

## Figures and Tables

**Table 1:** Hydrogen-bond geometry (Å, °)

*D*—H⋯*A*	*D*—H	H⋯*A*	*D*⋯*A*	*D*—H⋯*A*
O7—H7*D*⋯O8^i^	0.85	2.04	2.754 (6)	141
O7—H7*E*⋯O9^ii^	0.85	1.90	2.670 (9)	150
O8—H8*A*⋯O2^iii^	0.85	1.88	2.728 (8)	172
O8—H8*B*⋯N9^iv^	0.85	2.00	2.847 (10)	173
O9—H9*A*⋯O6^v^	0.85	1.82	2.665 (8)	170
O9—H9*B*⋯O10^vi^	0.85	2.02	2.858 (9)	171
O10—H10*A*⋯O8^vii^	0.85	2.07	2.915 (9)	175
O10—H10*B*⋯O2^iii^	0.85	2.14	2.988 (10)	175
C27—H27⋯O8^iv^	0.93	2.59	3.406 (11)	147
C29—H29*B*⋯O4^i^	0.96	2.57	3.363 (12)	140
C22—H22*A*⋯O7	0.96	2.52	3.147 (11)	123
C29—H29*C*⋯N7	0.96	2.49	2.874 (10)	104
C33—H33*B*⋯*Cg*^viii^	0.96	2.97	3.893	161

Symmetry codes: (i) 
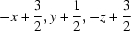
; (ii) 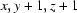; (iii) 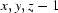; (iv) 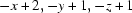; (v) 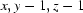; (vi) 
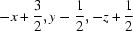
; (vii) 
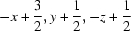
; (viii) 
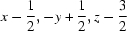
. Cg is the centroid of the N2,N3,C8–C10 ring.

**Fig. 1.**
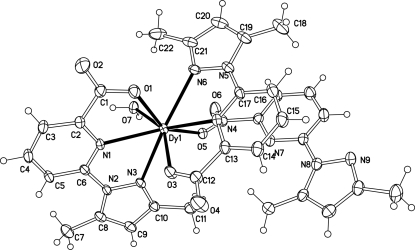
The molecular structure of the title compound showing 50% probability displacement ellipsoids and the atom-numbering scheme.

**Fig. 2.**
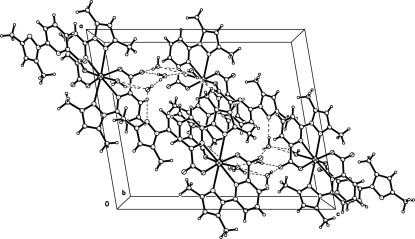
Crystal packing of the title compound showing the hydrogen bonding interactions as dashed lines.

## supplementary crystallographic information

### Comment

Recently we reported the crystal structures of bis[6-(3,5-dimethyl-1*H*-pyrazol-1-yl)picolinato]zinc(II) trihydrate (Yin *et al.*, 2007) and bis[3-chloro-6-(3,5-dimethyl-1*H*-pyrazol-1-yl)picolinato]nickel(II) tetrahydrate (Zhao *et al.*, 2008). The title compound consists of the central asymmetric mononuclear dysprosium(III) complex and three uncoordinated water molecules. The Dy atom is eight-coordinated by four N atoms and four O atoms derived from three tridentate, deprotonated 6-(3,5-dimethyl-1*H*-pyrazol-1-yl)picolinate ligands (DPP) and one water molecule, that define a pseudo-tricapped trigonal environment for the dysprosium atom. The angles around the Dy(III) atom range from 61.39 (18) to 155.29 (19)°, the Dy—O distances range between 2.235 (5) and 2.368 (5) Å, and the Dy—N distances range between 2.511 (6) and 2.598 (6) Å, *i.e.* normal values. The C1—C2, C12—C13 and C23—C24 bond lengths range between 1.515 (11) and 1.518 (10) Å, being in the normal C—C ranges found in dysprosium carboxylate complexes (Baggio *et al.* (2003) and literature cited therein).

In the crystal structure, the oxygen atoms contribute to the formation of intermolecular hydrogen bonds involving the solvate water molecules. Four water molecules, two DDP O atoms and one DDP N atom form rings *via* intermolecular H···O—H···O and H···O—H···N hydrogen bonds (Fig. 2). These interactions and two intermolecular C—H···O hydrogen bonds and a C—H···π contact (*Cg*^viii^ is the centroid of ring atoms N2, N3, C8, C9, C10) link the complexes into a three-dimensional network (Table 1). Two weak intramolecular C_methyl_—H···O/N hydrogen bonds contribute to the conformational stability of the title complex.

### Experimental

6-(3,5-dimethyl-1*H*-pyrazol-1-yl)picolinic acid, and Dy(NO_3_)_3_. 6H_2_O were available commercially and were used without further purification. Equimolar 6-(3,5-dimethyl-1*H*-pyrazol-1-yl)picolinic acid (1 mmol, 217 mg) was dissolved in anhydrous ethyl alcohol (AR,99.9%) (15 ml). The mixture was stirred to give a clear solution. To this solution was added Dy(NO_3_)_3_. 6H_2_O (0.33 mmol,151 mg) in anhydrous alcohol (10 ml). After keeping the resulting solution in air to evaporate about half of the solvents, colorless blocks of the title complex were formed. The crystals were isolated and washed with alcohol three times (Yield75%). Elemental analysis: found: C, 44.63; H, 4.42; N, 14.18; calc. for C_33_H_38_DyN_9_O_10_: C, 44.88; H, 4.34; N, 14.27.

### Refinement

H atoms on C atoms were positoned geometrically and refined using a riding model with C—H = 0.96 Å and *U*_iso_(H) = 1.2*U*_eq_(C). The water H atoms were located in difference Fourier maps and the O—H distances were constrained 0.85 Å, with *U*_iso_(H) = 1.2*U*_eq_(O).

### Crystal data

**Table:**

[Dy(C_11_H_10_N_3_O_2_)_3_(H_2_O)]·3H_2_O	*F*_000_ = 1780
*M**_r_* = 883.22	*D*_x_ = 1.615 Mg m^−^^3^
Monoclinic, *P*2_1_/*n*	Mo *K*α radiation λ = 0.71073 Å
Hall symbol: -P 2yn	Cell parameters from 5527 reflections
*a* = 15.4709 (18) Å	θ = 2.2–27.0º
*b* = 12.8466 (12) Å	µ = 2.13 mm^−^^1^
*c* = 18.543 (2) Å	*T* = 298 (2) K
β = 99.741 (2)º	Block, colorless
*V* = 3632.3 (7) Å^3^	0.30 × 0.24 × 0.17 mm
*Z* = 4	

### Data collection

**Table:**

Siemens CCD area-detector diffractometer	6277 independent reflections
Radiation source: fine-focus sealed tube	4009 reflections with *I* > 2σ(*I*)
Monochromator: graphite	*R*_int_ = 0.063
*T* = 298(2) K	θ_max_ = 25.0º
φ and ω scans	θ_min_ = 1.6º
Absorption correction: multi-scan(SADABS; Sheldrick, 1996)	*h* = −17→18
*T*_min_ = 0.568, *T*_max_ = 0.714	*k* = −15→11
17074 measured reflections	*l* = −20→22

### Refinement

**Table:**

Refinement on *F*^2^	Secondary atom site location: difference Fourier map
Least-squares matrix: full	Hydrogen site location: inferred from neighbouring sites
*R*[*F*^2^ > 2σ(*F*^2^)] = 0.050	H-atom parameters constrained
*wR*(*F*^2^) = 0.118	*w* = 1/[σ^2^(*F*_o_^2^) + (0.0405*P*)^2^ + 8.4683*P*] where *P* = (*F*_o_^2^ + 2*F*_c_^2^)/3
*S* = 1.04	(Δ/σ)_max_ = 0.001
6277 reflections	Δρ_max_ = 1.45 e Å^−^^3^
478 parameters	Δρ_min_ = −0.93 e Å^−^^3^
Primary atom site location: structure-invariant direct methods	Extinction correction: none

### Special details

**Table:**

Geometry. All e.s.d.'s (except the e.s.d. in the dihedral angle between two l.s. planes) are estimated using the full covariance matrix. The cell e.s.d.'s are taken into account individually in the estimation of e.s.d.'s in distances, angles and torsion angles; correlations between e.s.d.'s in cell parameters are only used when they are defined by crystal symmetry. An approximate (isotropic) treatment of cell e.s.d.'s is used for estimating e.s.d.'s involving l.s. planes.
Refinement. Refinement of *F*^2^ against ALL reflections. The weighted *R*-factor *wR* and goodness of fit *S* are based on *F*^2^, conventional *R*-factors *R* are based on *F*, with *F* set to zero for negative *F*^2^. The threshold expression of *F*^2^ > σ(*F*^2^) is used only for calculating *R*-factors(gt) *etc*. and is not relevant to the choice of reflections for refinement. *R*-factors based on *F*^2^ are statistically about twice as large as those based on *F*, and *R*- factors based on ALL data will be even larger.

### Fractional atomic coordinates and isotropic or equivalent isotropic displacement parameters (Å^2^)

**Table:**

	*x*	*y*	*z*	*U*_iso_*/*U*_eq_	
Dy1	0.75071 (2)	0.66687 (2)	1.003769 (16)	0.03410 (12)	
N1	0.6309 (4)	0.5937 (4)	1.0664 (3)	0.0361 (14)	
N2	0.5241 (4)	0.6864 (5)	0.9895 (3)	0.0396 (15)	
N3	0.5898 (4)	0.7133 (5)	0.9520 (3)	0.0386 (14)	
N4	0.8662 (4)	0.5887 (5)	0.9402 (3)	0.0414 (15)	
N5	0.9742 (4)	0.6866 (5)	1.0092 (4)	0.0506 (17)	
N6	0.9143 (4)	0.7066 (5)	1.0545 (3)	0.0485 (17)	
N7	0.8795 (4)	0.8013 (5)	0.8338 (3)	0.0455 (16)	
N8	0.9188 (4)	0.7169 (5)	0.7327 (3)	0.0485 (16)	
N9	0.9861 (5)	0.6876 (5)	0.6967 (3)	0.0524 (18)	
O1	0.7997 (4)	0.5476 (5)	1.0950 (3)	0.0616 (16)	
O2	0.7858 (4)	0.4614 (5)	1.1964 (3)	0.084 (2)	
O3	0.7008 (3)	0.5403 (4)	0.9230 (3)	0.0567 (15)	
O4	0.7111 (5)	0.4224 (6)	0.8381 (4)	0.094 (2)	
O5	0.7797 (3)	0.8105 (4)	0.9442 (2)	0.0444 (13)	
O6	0.8256 (5)	0.9737 (5)	0.9672 (4)	0.087 (2)	
O7	0.7206 (3)	0.7929 (4)	1.0893 (2)	0.0492 (13)	
H7D	0.6888	0.7698	1.1189	0.059*	
H7E	0.6992	0.8488	1.0692	0.059*	
O8	0.8288 (4)	0.2837 (4)	0.2749 (3)	0.0619 (15)	
H8A	0.8104	0.3374	0.2502	0.074*	
H8B	0.8844	0.2873	0.2855	0.074*	
O9	0.7072 (5)	−0.0051 (5)	0.0552 (3)	0.094 (3)	
H9A	0.7490	−0.0074	0.0308	0.113*	
H9B	0.7232	0.0315	0.0933	0.113*	
O10	0.7578 (5)	0.6130 (6)	0.3125 (3)	0.099 (2)	
H10A	0.7319	0.6644	0.2894	0.119*	
H10B	0.7670	0.5674	0.2814	0.119*	
C1	0.7554 (6)	0.5094 (7)	1.1399 (4)	0.054 (2)	
C2	0.6573 (5)	0.5279 (6)	1.1235 (4)	0.0439 (19)	
C3	0.6000 (6)	0.4839 (7)	1.1636 (4)	0.055 (2)	
H3	0.6197	0.4401	1.2029	0.066*	
C4	0.5124 (6)	0.5070 (7)	1.1436 (4)	0.059 (2)	
H4	0.4716	0.4759	1.1683	0.071*	
C5	0.4849 (5)	0.5744 (7)	1.0883 (4)	0.052 (2)	
H5	0.4259	0.5921	1.0761	0.063*	
C6	0.5470 (5)	0.6171 (6)	1.0500 (4)	0.0406 (18)	
C7	0.3586 (5)	0.7125 (8)	0.9814 (5)	0.069 (3)	
H7A	0.3401	0.6412	0.9763	0.103*	
H7B	0.3158	0.7560	0.9522	0.103*	
H7C	0.3644	0.7329	1.0318	0.103*	
C8	0.4452 (5)	0.7243 (7)	0.9564 (4)	0.049 (2)	
C9	0.4607 (5)	0.7774 (7)	0.8958 (4)	0.053 (2)	
H9	0.4196	0.8126	0.8621	0.064*	
C10	0.5501 (5)	0.7684 (6)	0.8948 (4)	0.047 (2)	
C11	0.5989 (6)	0.8110 (7)	0.8377 (4)	0.060 (2)	
H11A	0.6177	0.8808	0.8505	0.090*	
H11B	0.5610	0.8113	0.7910	0.090*	
H11C	0.6491	0.7682	0.8351	0.090*	
C12	0.7414 (6)	0.4911 (7)	0.8808 (4)	0.052 (2)	
C13	0.8373 (5)	0.5204 (6)	0.8873 (4)	0.048 (2)	
C14	0.8902 (6)	0.4777 (7)	0.8429 (4)	0.059 (2)	
H14	0.8681	0.4303	0.8065	0.071*	
C15	0.9773 (6)	0.5071 (7)	0.8537 (5)	0.063 (2)	
H15	1.0145	0.4802	0.8239	0.075*	
C16	1.0093 (6)	0.5766 (7)	0.9088 (5)	0.060 (2)	
H16	1.0680	0.5961	0.9174	0.072*	
C17	0.9503 (5)	0.6165 (6)	0.9511 (4)	0.046 (2)	
C18	1.1332 (6)	0.7357 (9)	0.9999 (6)	0.094 (4)	
H18A	1.1539	0.6657	0.9966	0.142*	
H18B	1.1781	0.7769	1.0288	0.142*	
H18C	1.1189	0.7649	0.9517	0.142*	
C19	1.0545 (6)	0.7351 (8)	1.0347 (5)	0.068 (3)	
C20	1.0409 (7)	0.7832 (8)	1.0969 (6)	0.080 (3)	
H20	1.0822	0.8231	1.1272	0.096*	
C21	0.9556 (6)	0.7636 (8)	1.1085 (5)	0.065 (3)	
C22	0.9154 (7)	0.7962 (9)	1.1728 (5)	0.091 (4)	
H22A	0.8691	0.8451	1.1573	0.136*	
H22B	0.9593	0.8280	1.2088	0.136*	
H22C	0.8919	0.7362	1.1937	0.136*	
C23	0.8286 (5)	0.8888 (7)	0.9366 (4)	0.0481 (19)	
C24	0.8961 (5)	0.8752 (6)	0.8868 (4)	0.0472 (19)	
C25	0.9700 (6)	0.9355 (7)	0.8983 (4)	0.060 (2)	
H25	0.9788	0.9847	0.9356	0.073*	
C26	1.0319 (6)	0.9207 (7)	0.8524 (5)	0.066 (3)	
H26	1.0837	0.9590	0.8592	0.080*	
C27	1.0154 (6)	0.8492 (7)	0.7972 (4)	0.060 (2)	
H27	1.0554	0.8389	0.7656	0.072*	
C28	0.9382 (5)	0.7923 (6)	0.7890 (4)	0.0464 (19)	
C29	0.7571 (6)	0.6856 (7)	0.7301 (4)	0.062 (2)	
H29A	0.7139	0.6385	0.7052	0.092*	
H29B	0.7382	0.7560	0.7195	0.092*	
H29C	0.7642	0.6737	0.7818	0.092*	
C30	0.8417 (6)	0.6681 (7)	0.7049 (4)	0.051 (2)	
C31	0.8611 (6)	0.6068 (7)	0.6500 (4)	0.062 (2)	
H31	0.8221	0.5636	0.6202	0.074*	
C32	0.9491 (6)	0.6201 (7)	0.6464 (4)	0.058 (2)	
C33	1.0021 (7)	0.5682 (8)	0.5948 (5)	0.081 (3)	
H33A	1.0631	0.5689	0.6163	0.121*	
H33B	0.9941	0.6054	0.5493	0.121*	
H33C	0.9828	0.4976	0.5862	0.121*	

### Atomic displacement parameters (Å^2^)

**Table:**

	*U*^11^	*U*^22^	*U*^33^	*U*^12^	*U*^13^	*U*^23^
Dy1	0.02830 (17)	0.03581 (18)	0.03982 (18)	−0.0007 (2)	0.01043 (12)	0.00066 (18)
N1	0.035 (4)	0.035 (4)	0.042 (3)	−0.007 (3)	0.015 (3)	0.001 (3)
N2	0.028 (3)	0.048 (4)	0.045 (3)	−0.004 (3)	0.013 (3)	−0.004 (3)
N3	0.024 (3)	0.045 (4)	0.048 (3)	0.003 (3)	0.009 (3)	−0.002 (3)
N4	0.041 (4)	0.041 (4)	0.046 (3)	0.002 (3)	0.016 (3)	0.003 (3)
N5	0.033 (4)	0.052 (5)	0.066 (4)	−0.006 (3)	0.005 (3)	0.005 (3)
N6	0.033 (4)	0.057 (5)	0.052 (4)	0.000 (3)	−0.003 (3)	0.003 (3)
N7	0.048 (4)	0.047 (4)	0.044 (3)	0.005 (3)	0.016 (3)	0.006 (3)
N8	0.051 (4)	0.050 (4)	0.046 (3)	0.006 (3)	0.015 (3)	0.001 (3)
N9	0.054 (5)	0.050 (5)	0.058 (4)	0.003 (3)	0.024 (3)	−0.002 (3)
O1	0.045 (4)	0.074 (4)	0.068 (3)	0.012 (3)	0.015 (3)	0.023 (3)
O2	0.077 (5)	0.092 (5)	0.082 (4)	0.023 (4)	0.011 (4)	0.050 (4)
O3	0.042 (4)	0.063 (4)	0.069 (3)	−0.011 (3)	0.019 (3)	−0.027 (3)
O4	0.070 (5)	0.096 (6)	0.121 (5)	−0.017 (4)	0.025 (4)	−0.061 (5)
O5	0.043 (3)	0.049 (3)	0.044 (3)	−0.007 (2)	0.013 (2)	0.003 (2)
O6	0.102 (6)	0.070 (5)	0.104 (5)	−0.026 (4)	0.061 (4)	−0.037 (4)
O7	0.055 (4)	0.052 (3)	0.046 (3)	−0.003 (3)	0.022 (2)	−0.003 (2)
O8	0.062 (4)	0.066 (4)	0.060 (3)	0.012 (3)	0.020 (3)	0.013 (3)
O9	0.135 (7)	0.073 (5)	0.093 (4)	−0.003 (4)	0.069 (5)	−0.015 (4)
O10	0.107 (7)	0.105 (6)	0.077 (4)	0.014 (5)	−0.007 (4)	0.001 (4)
C1	0.051 (6)	0.059 (6)	0.052 (4)	0.004 (4)	0.011 (4)	0.017 (4)
C2	0.044 (5)	0.043 (5)	0.045 (4)	−0.005 (4)	0.012 (3)	0.004 (3)
C3	0.062 (6)	0.060 (6)	0.046 (4)	−0.010 (5)	0.019 (4)	0.010 (4)
C4	0.058 (6)	0.069 (6)	0.056 (5)	−0.020 (5)	0.026 (4)	0.004 (5)
C5	0.037 (5)	0.066 (6)	0.057 (5)	−0.015 (4)	0.017 (4)	−0.002 (4)
C6	0.032 (5)	0.045 (5)	0.046 (4)	−0.007 (4)	0.010 (3)	−0.010 (3)
C7	0.035 (5)	0.081 (7)	0.093 (6)	0.013 (5)	0.017 (4)	−0.002 (5)
C8	0.032 (5)	0.053 (5)	0.062 (5)	0.000 (4)	0.008 (4)	−0.006 (4)
C9	0.033 (5)	0.056 (6)	0.068 (5)	0.004 (4)	0.001 (4)	0.005 (4)
C10	0.036 (5)	0.050 (5)	0.054 (4)	0.002 (4)	0.006 (4)	0.009 (4)
C11	0.050 (5)	0.067 (6)	0.061 (5)	0.003 (5)	0.002 (4)	0.020 (4)
C12	0.052 (5)	0.049 (5)	0.057 (4)	−0.002 (4)	0.015 (4)	−0.018 (4)
C13	0.044 (5)	0.052 (6)	0.053 (4)	0.008 (4)	0.019 (4)	−0.001 (4)
C14	0.060 (6)	0.060 (6)	0.062 (5)	0.012 (5)	0.025 (4)	−0.006 (4)
C15	0.056 (6)	0.069 (7)	0.069 (5)	0.017 (5)	0.026 (5)	0.002 (5)
C16	0.044 (6)	0.067 (7)	0.071 (5)	0.006 (5)	0.019 (4)	0.012 (5)
C17	0.036 (5)	0.049 (5)	0.056 (4)	0.001 (4)	0.014 (4)	0.018 (4)
C18	0.047 (7)	0.100 (9)	0.135 (9)	−0.011 (6)	0.013 (6)	0.012 (7)
C19	0.035 (6)	0.073 (7)	0.092 (7)	−0.009 (5)	0.000 (5)	0.007 (6)
C20	0.052 (7)	0.075 (8)	0.103 (8)	−0.007 (6)	−0.017 (6)	−0.004 (6)
C21	0.045 (6)	0.068 (7)	0.075 (6)	−0.005 (5)	−0.013 (5)	0.000 (5)
C22	0.077 (8)	0.107 (9)	0.077 (6)	0.013 (7)	−0.018 (6)	−0.029 (6)
C23	0.046 (5)	0.053 (5)	0.047 (4)	−0.004 (4)	0.015 (4)	−0.002 (4)
C24	0.052 (5)	0.047 (5)	0.043 (4)	−0.004 (4)	0.011 (4)	0.004 (4)
C25	0.059 (6)	0.064 (6)	0.061 (5)	−0.011 (5)	0.019 (4)	−0.005 (4)
C26	0.062 (6)	0.066 (7)	0.075 (6)	−0.013 (5)	0.022 (5)	−0.008 (5)
C27	0.060 (6)	0.065 (6)	0.062 (5)	−0.008 (5)	0.028 (4)	−0.006 (4)
C28	0.049 (5)	0.048 (5)	0.045 (4)	0.003 (4)	0.015 (4)	0.005 (3)
C29	0.051 (5)	0.073 (7)	0.060 (5)	0.001 (5)	0.008 (4)	0.000 (4)
C30	0.058 (5)	0.054 (5)	0.042 (4)	0.008 (4)	0.011 (3)	0.006 (4)
C31	0.073 (7)	0.055 (6)	0.055 (5)	0.004 (5)	0.009 (4)	−0.001 (4)
C32	0.067 (6)	0.051 (6)	0.059 (5)	0.007 (5)	0.022 (5)	−0.001 (4)
C33	0.093 (8)	0.070 (7)	0.088 (6)	0.001 (6)	0.041 (6)	−0.023 (6)

### Geometric parameters (Å, °)

**Table:**

Dy1—O5	2.235 (5)	C7—H7A	0.9600
Dy1—O3	2.256 (5)	C7—H7B	0.9600
Dy1—O1	2.313 (5)	C7—H7C	0.9600
Dy1—O7	2.368 (5)	C8—C9	1.370 (11)
Dy1—N4	2.511 (6)	C9—C10	1.391 (11)
Dy1—N1	2.530 (5)	C9—H9	0.9300
Dy1—N3	2.582 (6)	C10—C11	1.504 (10)
Dy1—N6	2.598 (6)	C11—H11A	0.9600
N1—C6	1.317 (9)	C11—H11B	0.9600
N1—C2	1.362 (8)	C11—H11C	0.9600
N2—C8	1.360 (9)	C12—C13	1.515 (11)
N2—N3	1.369 (7)	C13—C14	1.371 (10)
N2—C6	1.429 (9)	C14—C15	1.380 (12)
N3—C10	1.335 (9)	C14—H14	0.9300
N4—C17	1.331 (9)	C15—C16	1.384 (12)
N4—C13	1.337 (9)	C15—H15	0.9300
N5—N6	1.375 (8)	C16—C17	1.396 (11)
N5—C19	1.399 (11)	C16—H16	0.9300
N5—C17	1.405 (10)	C18—C19	1.470 (12)
N6—C21	1.315 (10)	C18—H18A	0.9600
N7—C28	1.335 (9)	C18—H18B	0.9600
N7—C24	1.358 (9)	C18—H18C	0.9600
N8—C30	1.369 (10)	C19—C20	1.356 (13)
N8—N9	1.380 (8)	C20—C21	1.395 (13)
N8—C28	1.418 (9)	C20—H20	0.9300
N9—C32	1.331 (10)	C21—C22	1.496 (12)
O1—C1	1.262 (9)	C22—H22A	0.9600
O2—C1	1.239 (9)	C22—H22B	0.9600
O3—C12	1.254 (8)	C22—H22C	0.9600
O4—C12	1.226 (9)	C23—C24	1.518 (10)
O5—C23	1.280 (9)	C24—C25	1.368 (11)
O6—C23	1.233 (9)	C25—C26	1.399 (11)
O7—H7D	0.8499	C25—H25	0.9300
O7—H7E	0.8501	C26—C27	1.365 (11)
O8—H8A	0.8500	C26—H26	0.9300
O8—H8B	0.8500	C27—C28	1.388 (11)
O9—H9A	0.8499	C27—H27	0.9300
O9—H9B	0.8500	C29—C30	1.479 (11)
O10—H10A	0.8500	C29—H29A	0.9600
O10—H10B	0.8499	C29—H29B	0.9600
C1—C2	1.515 (11)	C29—H29C	0.9600
C2—C3	1.371 (10)	C30—C31	1.361 (10)
C3—C4	1.377 (12)	C31—C32	1.385 (12)
C3—H3	0.9300	C31—H31	0.9300
C4—C5	1.355 (11)	C32—C33	1.516 (11)
C4—H4	0.9300	C33—H33A	0.9600
C5—C6	1.399 (10)	C33—H33B	0.9600
C5—H5	0.9300	C33—H33C	0.9600
C7—C8	1.498 (10)		
			
O5—Dy1—O3	109.97 (18)	C10—C9—H9	126.8
O5—Dy1—O1	147.2 (2)	N3—C10—C9	111.0 (7)
O3—Dy1—O1	92.3 (2)	N3—C10—C11	122.3 (7)
O5—Dy1—O7	81.11 (18)	C9—C10—C11	126.7 (7)
O3—Dy1—O7	148.61 (19)	C10—C11—H11A	109.5
O1—Dy1—O7	92.30 (19)	C10—C11—H11B	109.5
O5—Dy1—N4	83.08 (18)	H11A—C11—H11B	109.5
O3—Dy1—N4	65.86 (19)	C10—C11—H11C	109.5
O1—Dy1—N4	84.69 (19)	H11A—C11—H11C	109.5
O7—Dy1—N4	145.5 (2)	H11B—C11—H11C	109.5
O5—Dy1—N1	139.29 (19)	O4—C12—O3	126.1 (8)
O3—Dy1—N1	80.91 (18)	O4—C12—C13	119.5 (7)
O1—Dy1—N1	65.95 (19)	O3—C12—C13	114.3 (7)
O7—Dy1—N1	72.77 (17)	N4—C13—C14	123.0 (8)
N4—Dy1—N1	134.60 (18)	N4—C13—C12	115.0 (6)
O5—Dy1—N3	83.51 (18)	C14—C13—C12	122.0 (8)
O3—Dy1—N3	73.73 (19)	C13—C14—C15	118.0 (9)
O1—Dy1—N3	126.92 (19)	C13—C14—H14	121.0
O7—Dy1—N3	78.72 (18)	C15—C14—H14	121.0
N4—Dy1—N3	129.51 (18)	C14—C15—C16	120.2 (8)
N1—Dy1—N3	61.39 (18)	C14—C15—H15	119.9
O5—Dy1—N6	75.63 (19)	C16—C15—H15	119.9
O3—Dy1—N6	125.8 (2)	C15—C16—C17	117.7 (8)
O1—Dy1—N6	71.8 (2)	C15—C16—H16	121.2
O7—Dy1—N6	85.02 (19)	C17—C16—H16	121.2
N4—Dy1—N6	61.4 (2)	N4—C17—C16	122.1 (8)
N1—Dy1—N6	130.68 (19)	N4—C17—N5	114.2 (7)
N3—Dy1—N6	155.29 (19)	C16—C17—N5	123.6 (8)
C6—N1—C2	118.4 (6)	C19—C18—H18A	109.5
C6—N1—Dy1	125.4 (5)	C19—C18—H18B	109.5
C2—N1—Dy1	116.1 (5)	H18A—C18—H18B	109.5
C8—N2—N3	111.6 (6)	C19—C18—H18C	109.5
C8—N2—C6	131.4 (6)	H18A—C18—H18C	109.5
N3—N2—C6	116.7 (6)	H18B—C18—H18C	109.5
C10—N3—N2	104.8 (6)	C20—C19—N5	103.5 (8)
C10—N3—Dy1	134.6 (5)	C20—C19—C18	129.0 (10)
N2—N3—Dy1	120.3 (4)	N5—C19—C18	127.5 (9)
C17—N4—C13	119.0 (6)	C19—C20—C21	109.6 (9)
C17—N4—Dy1	125.3 (5)	C19—C20—H20	125.2
C13—N4—Dy1	115.5 (5)	C21—C20—H20	125.2
N6—N5—C19	111.2 (7)	N6—C21—C20	109.5 (9)
N6—N5—C17	118.1 (6)	N6—C21—C22	123.3 (9)
C19—N5—C17	130.3 (8)	C20—C21—C22	127.2 (9)
C21—N6—N5	106.2 (7)	C21—C22—H22A	109.5
C21—N6—Dy1	134.6 (6)	C21—C22—H22B	109.5
N5—N6—Dy1	117.3 (4)	H22A—C22—H22B	109.5
C28—N7—C24	116.5 (7)	C21—C22—H22C	109.5
C30—N8—N9	112.0 (6)	H22A—C22—H22C	109.5
C30—N8—C28	130.6 (7)	H22B—C22—H22C	109.5
N9—N8—C28	117.4 (7)	O6—C23—O5	125.2 (7)
C32—N9—N8	104.3 (7)	O6—C23—C24	117.7 (7)
C1—O1—Dy1	126.3 (5)	O5—C23—C24	117.1 (7)
C12—O3—Dy1	128.8 (5)	N7—C24—C25	124.0 (7)
C23—O5—Dy1	151.0 (5)	N7—C24—C23	117.1 (7)
Dy1—O7—H7D	113.2	C25—C24—C23	118.8 (7)
Dy1—O7—H7E	112.9	C24—C25—C26	117.9 (8)
H7D—O7—H7E	110.6	C24—C25—H25	121.0
H8A—O8—H8B	108.2	C26—C25—H25	121.0
H9A—O9—H9B	108.6	C27—C26—C25	119.1 (9)
H10A—O10—H10B	108.3	C27—C26—H26	120.4
O2—C1—O1	125.5 (8)	C25—C26—H26	120.4
O2—C1—C2	118.1 (7)	C26—C27—C28	119.0 (8)
O1—C1—C2	116.4 (7)	C26—C27—H27	120.5
N1—C2—C3	122.7 (8)	C28—C27—H27	120.5
N1—C2—C1	114.3 (6)	N7—C28—C27	123.3 (7)
C3—C2—C1	123.0 (7)	N7—C28—N8	115.9 (7)
C2—C3—C4	117.6 (8)	C27—C28—N8	120.8 (7)
C2—C3—H3	121.2	C30—C29—H29A	109.5
C4—C3—H3	121.2	C30—C29—H29B	109.5
C5—C4—C3	120.7 (7)	H29A—C29—H29B	109.5
C5—C4—H4	119.7	C30—C29—H29C	109.5
C3—C4—H4	119.7	H29A—C29—H29C	109.5
C4—C5—C6	118.7 (8)	H29B—C29—H29C	109.5
C4—C5—H5	120.6	C31—C30—N8	104.9 (8)
C6—C5—H5	120.6	C31—C30—C29	130.0 (9)
N1—C6—C5	121.8 (7)	N8—C30—C29	125.0 (7)
N1—C6—N2	115.3 (6)	C30—C31—C32	107.9 (8)
C5—C6—N2	122.8 (7)	C30—C31—H31	126.0
C8—C7—H7A	109.5	C32—C31—H31	126.0
C8—C7—H7B	109.5	N9—C32—C31	110.9 (7)
H7A—C7—H7B	109.5	N9—C32—C33	120.9 (9)
C8—C7—H7C	109.5	C31—C32—C33	128.2 (9)
H7A—C7—H7C	109.5	C32—C33—H33A	109.5
H7B—C7—H7C	109.5	C32—C33—H33B	109.5
N2—C8—C9	106.2 (7)	H33A—C33—H33B	109.5
N2—C8—C7	127.3 (7)	C32—C33—H33C	109.5
C9—C8—C7	126.5 (8)	H33A—C33—H33C	109.5
C8—C9—C10	106.4 (7)	H33B—C33—H33C	109.5
C8—C9—H9	126.8		
			
O5—Dy1—N1—C6	28.1 (7)	O2—C1—C2—C3	−7.8 (13)
O3—Dy1—N1—C6	−82.1 (6)	O1—C1—C2—C3	174.3 (8)
O1—Dy1—N1—C6	−178.8 (6)	N1—C2—C3—C4	1.1 (12)
O7—Dy1—N1—C6	80.6 (6)	C1—C2—C3—C4	179.8 (8)
N4—Dy1—N1—C6	−124.4 (5)	C2—C3—C4—C5	−2.9 (13)
N3—Dy1—N1—C6	−5.7 (5)	C3—C4—C5—C6	2.6 (13)
N6—Dy1—N1—C6	147.9 (5)	C2—N1—C6—C5	−1.5 (11)
O5—Dy1—N1—C2	−149.3 (4)	Dy1—N1—C6—C5	−178.8 (5)
O3—Dy1—N1—C2	100.5 (5)	C2—N1—C6—N2	−180.0 (6)
O1—Dy1—N1—C2	3.9 (5)	Dy1—N1—C6—N2	2.7 (9)
O7—Dy1—N1—C2	−96.8 (5)	C4—C5—C6—N1	−0.3 (12)
N4—Dy1—N1—C2	58.2 (6)	C4—C5—C6—N2	178.0 (7)
N3—Dy1—N1—C2	176.9 (5)	C8—N2—C6—N1	177.8 (7)
N6—Dy1—N1—C2	−29.5 (6)	N3—N2—C6—N1	5.3 (9)
C8—N2—N3—C10	−0.3 (8)	C8—N2—C6—C5	−0.6 (12)
C6—N2—N3—C10	173.7 (6)	N3—N2—C6—C5	−173.1 (6)
C8—N2—N3—Dy1	175.3 (5)	N3—N2—C8—C9	−0.1 (9)
C6—N2—N3—Dy1	−10.7 (8)	C6—N2—C8—C9	−172.9 (7)
O5—Dy1—N3—C10	23.7 (7)	N3—N2—C8—C7	−179.1 (8)
O3—Dy1—N3—C10	−89.3 (7)	C6—N2—C8—C7	8.1 (14)
O1—Dy1—N3—C10	−169.7 (7)	N2—C8—C9—C10	0.4 (9)
O7—Dy1—N3—C10	105.9 (7)	C7—C8—C9—C10	179.4 (8)
N4—Dy1—N3—C10	−51.7 (8)	N2—N3—C10—C9	0.5 (9)
N1—Dy1—N3—C10	−177.7 (8)	Dy1—N3—C10—C9	−174.2 (5)
N6—Dy1—N3—C10	56.1 (9)	N2—N3—C10—C11	−178.3 (7)
O5—Dy1—N3—N2	−150.3 (5)	Dy1—N3—C10—C11	7.0 (12)
O3—Dy1—N3—N2	96.7 (5)	C8—C9—C10—N3	−0.6 (10)
O1—Dy1—N3—N2	16.2 (6)	C8—C9—C10—C11	178.2 (8)
O7—Dy1—N3—N2	−68.1 (5)	Dy1—O3—C12—O4	179.7 (7)
N4—Dy1—N3—N2	134.2 (4)	Dy1—O3—C12—C13	1.6 (11)
N1—Dy1—N3—N2	8.3 (4)	C17—N4—C13—C14	−0.5 (12)
N6—Dy1—N3—N2	−117.9 (6)	Dy1—N4—C13—C14	174.0 (6)
O5—Dy1—N4—C17	64.5 (6)	C17—N4—C13—C12	178.1 (7)
O3—Dy1—N4—C17	−180.0 (6)	Dy1—N4—C13—C12	−7.4 (8)
O1—Dy1—N4—C17	−85.0 (6)	O4—C12—C13—N4	−173.8 (8)
O7—Dy1—N4—C17	1.3 (7)	O3—C12—C13—N4	4.4 (11)
N1—Dy1—N4—C17	−133.2 (5)	O4—C12—C13—C14	4.8 (13)
N3—Dy1—N4—C17	140.1 (5)	O3—C12—C13—C14	−177.0 (8)
N6—Dy1—N4—C17	−12.9 (5)	N4—C13—C14—C15	0.0 (13)
O5—Dy1—N4—C13	−109.6 (5)	C12—C13—C14—C15	−178.5 (8)
O3—Dy1—N4—C13	5.9 (5)	C13—C14—C15—C16	1.0 (13)
O1—Dy1—N4—C13	100.9 (5)	C14—C15—C16—C17	−1.3 (13)
O7—Dy1—N4—C13	−172.8 (5)	C13—N4—C17—C16	0.1 (11)
N1—Dy1—N4—C13	52.7 (6)	Dy1—N4—C17—C16	−173.8 (5)
N3—Dy1—N4—C13	−34.0 (6)	C13—N4—C17—N5	−178.5 (6)
N6—Dy1—N4—C13	173.0 (6)	Dy1—N4—C17—N5	7.6 (9)
C19—N5—N6—C21	−1.7 (9)	C15—C16—C17—N4	0.8 (12)
C17—N5—N6—C21	172.6 (7)	C15—C16—C17—N5	179.3 (7)
C19—N5—N6—Dy1	164.8 (5)	N6—N5—C17—N4	9.5 (10)
C17—N5—N6—Dy1	−20.8 (8)	C19—N5—C17—N4	−177.4 (8)
O5—Dy1—N6—C21	88.3 (8)	N6—N5—C17—C16	−169.1 (7)
O3—Dy1—N6—C21	−167.2 (8)	C19—N5—C17—C16	3.9 (13)
O1—Dy1—N6—C21	−87.9 (8)	N6—N5—C19—C20	0.8 (10)
O7—Dy1—N6—C21	6.2 (8)	C17—N5—C19—C20	−172.6 (8)
N4—Dy1—N6—C21	178.2 (9)	N6—N5—C19—C18	−177.3 (9)
N1—Dy1—N6—C21	−55.9 (9)	C17—N5—C19—C18	9.3 (16)
N3—Dy1—N6—C21	55.0 (10)	N5—C19—C20—C21	0.4 (11)
O5—Dy1—N6—N5	−73.4 (5)	C18—C19—C20—C21	178.4 (10)
O3—Dy1—N6—N5	31.1 (6)	N5—N6—C21—C20	1.9 (10)
O1—Dy1—N6—N5	110.5 (5)	Dy1—N6—C21—C20	−161.2 (6)
O7—Dy1—N6—N5	−155.5 (5)	N5—N6—C21—C22	−175.6 (9)
N4—Dy1—N6—N5	16.5 (5)	Dy1—N6—C21—C22	21.3 (14)
N1—Dy1—N6—N5	142.4 (5)	C19—C20—C21—N6	−1.5 (12)
N3—Dy1—N6—N5	−106.7 (6)	C19—C20—C21—C22	175.9 (10)
C30—N8—N9—C32	0.1 (8)	Dy1—O5—C23—O6	−86.0 (14)
C28—N8—N9—C32	−178.2 (7)	Dy1—O5—C23—C24	93.2 (10)
O5—Dy1—O1—C1	138.2 (6)	C28—N7—C24—C25	−3.3 (12)
O3—Dy1—O1—C1	−87.9 (7)	C28—N7—C24—C23	178.1 (7)
O7—Dy1—O1—C1	61.1 (7)	O6—C23—C24—N7	−156.2 (8)
N4—Dy1—O1—C1	−153.4 (7)	O5—C23—C24—N7	24.5 (10)
N1—Dy1—O1—C1	−8.9 (7)	O6—C23—C24—C25	25.1 (12)
N3—Dy1—O1—C1	−16.5 (8)	O5—C23—C24—C25	−154.1 (8)
N6—Dy1—O1—C1	145.1 (7)	N7—C24—C25—C26	0.6 (13)
O5—Dy1—O3—C12	68.4 (7)	C23—C24—C25—C26	179.2 (8)
O1—Dy1—O3—C12	−87.1 (7)	C24—C25—C26—C27	1.5 (14)
O7—Dy1—O3—C12	174.6 (6)	C25—C26—C27—C28	−0.9 (14)
N4—Dy1—O3—C12	−4.0 (7)	C24—N7—C28—C27	4.1 (11)
N1—Dy1—O3—C12	−152.3 (7)	C24—N7—C28—N8	−178.2 (6)
N3—Dy1—O3—C12	145.0 (7)	C26—C27—C28—N7	−2.1 (13)
N6—Dy1—O3—C12	−18.0 (8)	C26—C27—C28—N8	−179.7 (8)
O3—Dy1—O5—C23	−137.7 (10)	C30—N8—C28—N7	17.3 (12)
O1—Dy1—O5—C23	−7.7 (12)	N9—N8—C28—N7	−164.8 (6)
O7—Dy1—O5—C23	72.7 (10)	C30—N8—C28—C27	−164.9 (8)
N4—Dy1—O5—C23	−76.6 (10)	N9—N8—C28—C27	13.0 (11)
N1—Dy1—O5—C23	122.8 (10)	N9—N8—C30—C31	−0.2 (9)
N3—Dy1—O5—C23	152.2 (10)	C28—N8—C30—C31	177.8 (7)
N6—Dy1—O5—C23	−14.4 (10)	N9—N8—C30—C29	−177.9 (7)
Dy1—O1—C1—O2	−165.7 (7)	C28—N8—C30—C29	0.0 (13)
Dy1—O1—C1—C2	12.1 (11)	N8—C30—C31—C32	0.2 (9)
C6—N1—C2—C3	1.1 (11)	C29—C30—C31—C32	177.8 (8)
Dy1—N1—C2—C3	178.7 (6)	N8—N9—C32—C31	0.0 (9)
C6—N1—C2—C1	−177.7 (7)	N8—N9—C32—C33	−179.6 (7)
Dy1—N1—C2—C1	−0.1 (8)	C30—C31—C32—N9	−0.2 (10)
O2—C1—C2—N1	171.0 (8)	C30—C31—C32—C33	179.5 (8)
O1—C1—C2—N1	−6.9 (11)		

### Hydrogen-bond geometry (Å, °)

**Table:**

*D*—H···*A*	*D*—H	H···*A*	*D*···*A*	*D*—H···*A*
O7—H7D···O8^i^	0.85	2.04	2.754 (6)	141
O7—H7E···O9^ii^	0.85	1.90	2.670 (9)	150
O8—H8A···O2^iii^	0.85	1.88	2.728 (8)	172
O8—H8B···N9^iv^	0.85	2.00	2.847 (10)	173
O9—H9A···O6^v^	0.85	1.82	2.665 (8)	170
O9—H9B···O10^vi^	0.85	2.02	2.858 (9)	171
O10—H10A···O8^vii^	0.85	2.07	2.915 (9)	175
O10—H10B···O2^iii^	0.85	2.14	2.988 (10)	175
C27—H27···O8^iv^	0.93	2.59	3.406 (11)	147
C29—H29B···O4^i^	0.96	2.57	3.363 (12)	140
C22—H22A···O7	0.96	2.52	3.147 (11)	123
C29—H29C···N7	0.96	2.49	2.874 (10)	104
C33—H33B···Cg^viii^	0.96	2.97	3.893	161

Symmetry codes: (i) −*x*+3/2, *y*+1/2, −*z*+3/2; (ii) *x*, *y*+1, *z*+1; (iii) *x*, *y*, *z*−1; (iv) −*x*+2, −*y*+1, −*z*+1; (v) *x*, *y*−1, *z*−1; (vi) −*x*+3/2, *y*−1/2, −*z*+1/2; (vii) −*x*+3/2, *y*+1/2, −*z*+1/2;  (viii) *x*−1/2, −*y*+1/2, *z*−3/2.
